# Finite element analysis relating shape, material properties, and dimensions of taenioglossan radular teeth with trophic specialisations in Paludomidae (Gastropoda)

**DOI:** 10.1038/s41598-021-02102-8

**Published:** 2021-11-23

**Authors:** Wencke Krings, Jordi Marcé-Nogué, Stanislav N. Gorb

**Affiliations:** 1 Department of Mammalogy and Paleoanthropology, Center of Natural History (CeNak), Universität Hamburg, Martin-Luther-King-Platz 3, 20146 Hamburg, Germany; 2 Department of Functional Morphology and Biomechanics, Zoological Institute, Christian-Albrechts-Universität zu Kiel, Am Botanischen Garten 9, 24118 Kiel, Germany; 3 Department of Mechanical Engineering, Universitat Rovira i Virgili, Tarragona, Spain; 4 Institut Català de Paleontologia Miquel Crusafont, Universitat Autònoma de Barcelona, Cerdanyola del Vallès, Barcelona Spain

**Keywords:** Structural biology, Biomechanics

## Abstract

The radula, a chitinous membrane with embedded tooth rows, is the molluscan autapomorphy for feeding. The morphologies, arrangements and mechanical properties of teeth can vary between taxa, which is usually interpreted as adaptation to food. In previous studies, we proposed about trophic and other functional specialisations in taenioglossan radulae from species of African paludomid gastropods. These were based on the analysis of shape, material properties, force-resistance, and the mechanical behaviour of teeth, when interacting with an obstacle. The latter was previously simulated for one species (*Spekia zonata*) by the finite-element-analysis (FEA) and, for more species, observed in experiments. In the here presented work we test the previous hypotheses by applying the FEA on 3D modelled radulae, with incorporated material properties, from three additional paludomid species. These species forage either on algae attached to rocks (*Lavigeria grandis*), covering sand (*Cleopatra johnstoni*), or attached to plant surface and covering sand (*Bridouxia grandidieriana*). Since the analysed radulae vary greatly in their general size (e.g. width) and size of teeth between species, we additionally aimed at relating the simulated stress and strain distributions with the tooth sizes by altering the force/volume. For this purpose, we also included *S. zonata* again in the present study. Our FEA results show that smaller radulae are more affected by stress and strain than larger ones, when each tooth is loaded with the same force. However, the results are not fully in congruence with results from the previous breaking stress experiments, indicating that besides the parameter size, more mechanisms leading to reduced stress/strain must be present in radulae.

## Introduction

### The molluscan autapomorphy for feeding, the radula

**Figure 1:**
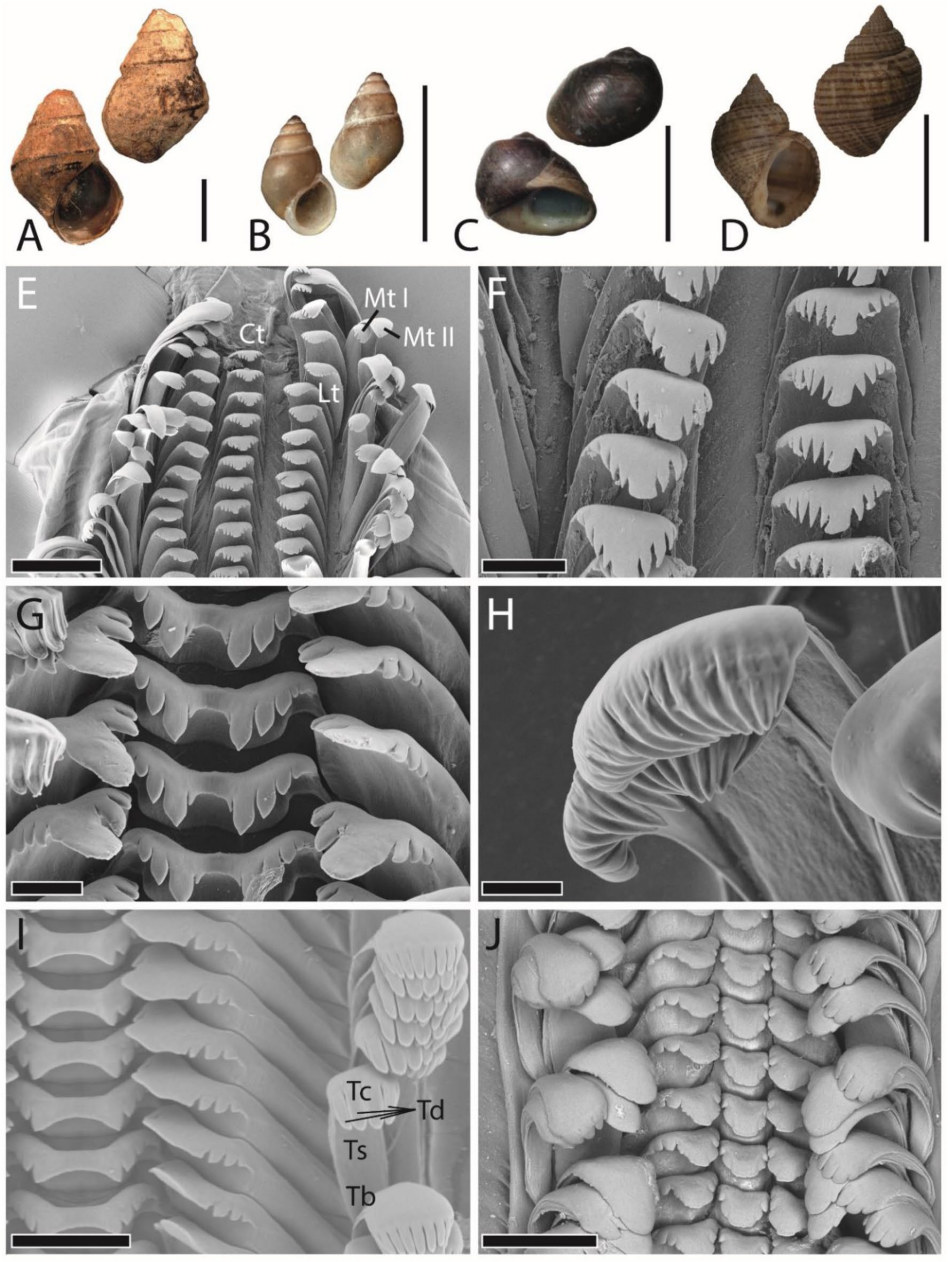
(**A**–**D**) Shells of adult specimens (adapted from^19,20^). (**A**) *Cleopatra johnstoni*, (**B**) *Bridouxia grandidieriana*, (**C**) *Spekia zonata*, (**D**) *Lavigeria grandis*. (**E**–**J**) SEM images of the working zone from adult specimens of (**E**–**F**) *C. johnstoni*, (**G**–**H**) *B. grandidieriana*, (**I**) *S. zonata*, and (**J**) *L. grandis* (**G** and **J** adapted from^19,20^). Ct = central tooth, Lt = lateral tooth, Mt I = marginal tooth I (inner marginal tooth), Mt II = marginal tooth II (outer marginal tooth), TB = tooth basis, Tc = tooth cusp, Td = tooth denticles, Ts = tooth stylus. Scale bars: A–B = 5 mm, C = 10 mm, F = 20 mm, E, J = 200 µm, F = 60 µm, G = 20 µm, H = 8 µm, I = 100 µm.

The radula is the molluscan autapomorphy for food gathering and processing. It consists of a chitinous membrane with embedded rows of teeth, which interact with the preferred food and the substrate the food is attached to (feeding substrate). Underlain odontophoral cartilages mechanically support the thin radula. Buccal mass muscles move it during foraging, resulting in shearing, cutting, and gathering actions^1–6^. Teeth usually have contact with the ingesta at their tooth cusps, which can be situated on elongated styli; these are connected to the radular membrane by their tooth bases (see Fig. 1). The tight interaction of teeth with the ingesta leads to some tooth wear (e.g.^7–12^), but the radula is constantly secreted and maturated by overlain epithelia in its posterior parts, the radular sack and formation zone (e.g.^13–18^). Then, mature teeth enter the radular anterior part, the working zone, where they are in use. Afterwards teeth eventually break loose in the degenerative zone and are probably digested.

### Functional and trophic specialisations of radular teeth

As the radula is the main structure used for the acquisition of food and the teeth represent the interface between organism and environment, the tooth shapes can reflect trophic adaptations (e.g. pointed teeth in carnivorous species). Additionally, functional specialisations of tooth types (e.g. loosening of food items, collecting particles, reinforcement of the radula, etc.) can be detected. Both types of specialisations are related to one another and can be reflected by radular morphology and structure (e.g.^12,21–42^) and by tooth material properties (i.e. hardness and Young’s modulus) (e.g.^5,9, 19,41,43–51^). Gradients in the mechanical properties hardness and Young’s modulus can contribute to the functionality of a structure by e.g. distributing stress, enabling deformation or the resistance to structural failure (e.g.^52–57^) and are present in teeth of chitons, limpets^44,46,58^, and paludomid gastropods^49–51^.

### Model system African Paludomidae

The Paludomidae from Lake Tanganyika and surrounding water bodies are representatives of a species flock, that is potentially the result of an adaptive radiation accompanied by trophic specialisation (for hypotheses on paludomid evolution see e.g.^19,41,59–68^). They possess taenioglossan radulae (seven teeth per row: one central, two laterals, two inner and two outer marginals) that show a great interspecific morphological diversity (see e.g.^19,41,68,69^). The species of the flock, which were on focus in previous studies on trophic specialisation, are substrate-specific and feed algae either from soft (sandy or muddy substrate), mixed (plant surface, sand, mud, and/or rock), or solid substrate (rocks)^19,41,51^. Some tooth shapes^19,41^ and the size and thickness of the tooth’s attachment with the underlain radular membrane^42^ were previously analysed and identified as adaptations to the preferred feeding substrate. Additionally, some taxa (all mixed and solid substrate feeders; e.g. *Bridouxia grandidieriana*, *Lavigeria grandis*, *Spekia zonata*) exhibit material property gradients along their teeth, with the cusp as the stiffest and hardest part, followed by the stylus, and finally the basis^49,51^. We additionally detected that soft substrate feeders (e.g. *Cleopatra johnstoni*) possess teeth with rather homogeneous material properties; thus the existence or absence of large-scaled gradients were previously also identified as adaptations^51^.

### Mechanical behaviour of teeth

The mechanical behaviour of structures can be computer-simulated and visualized by the finite-element-analysis (FEA), a software-based virtual method that solves mechanical problems. Here, bodies with defined material properties can be tested under the action of outer forces, resulting in the visualization of the deformation and distribution of stress and strain. This method was previously employed on various biological objects as a very useful approach in ecomorphological analyses of food processing structures (e.g.^70–82^).

FEA on radulae has been previously conducted on the dominant lateral tooth of the docoglossan radulae from polyplacophoran species^7,58^ and gastropod *Patella*^7^, on the isodont radula from the gastropod *Euhadra*^83^, and on the taenioglossan radula from the gastropod *Spekia zonata*^50^. The past stress and strain simulations, leading to hypotheses on the functional significance of the local material properties (deformation, transmission of stress, reduction of abrasion) and also tooth function, are either based on 2D shape and material property gradients^7^, on 3D shape with a homogeneous material^83^, or on 3D shape including material property gradients^50,58^. In our previous FEA approach on the paludomid *S. zonata,* we were able to identify the functional relationship between the shape and properties: we tested the properties’ effect on the stress and strain distribution by excluding or including gradients in the tooth FEA-model^50^. Hereby we detected that heterogeneous teeth (with gradients in Young’s modulus) have a higher capability of bending and transferring forces than homogeneous teeth (without gradients in Young’s modulus).

The proposed mechanical behaviour of *S. zonata*’s teeth under load has been later subsequently verified in the breaking stress experiments, where shear load was applied to individual tooth cusps of wet and dry radulae with a needle^20^. Here the force, needed to break teeth, and the behaviour of teeth (bending, twisting, and relying on other teeth) could be documented. Tooth failure under wet (native) condition usually occurred at two sites: (a) the long and slender outer teeth (marginal teeth) failed at their softest and most flexible part, at the stylus close to the basis. (b) The short and relatively stiff inner teeth (central and lateral teeth) relied on the adjacent teeth and did not break until the radular membrane ripped off. The cusps as the hardest and stiffest parts were not as prone to failure. These real experiments verified the previous computer based FEA simulations on *S. zonata*, displaying high values of stress in the marginal styli close to the bases, whereas the harder and stiffer marginal tooth cusps, the centrals, and the laterals were almost not affected by the stress under load^50^. Additionally, we discovered by these breaking stress experiments, that significantly higher force was needed to break wet teeth than dry ones. This was due to the interaction between adjacent teeth of the same type: wet teeth were capable of relying on teeth of adjacent rows^20,84^, enabled by mechanical property gradients (see also^49,51^), morphology (see also^41,50^), and their embedment in the radular membrane (see also^42^), leading to a higher force resistance due to a ‘collective effect’. Several previous hypotheses, proposed after examination of mounted radulae with scanning-electron-microscope (SEM), on the functional significance of the interaction of radular parts resulting in a proper stress distribution^42,85–90^ have been confirmed by our biomechanical experiments. A broader taxon sampling of paludomid gastropods (e.g. *Lavigeria grandis*, *Cleopatra johnstoni*, *Bridouxia grandidieriana*) in further breaking stress experiments demonstrated, that the degree, to which this ‘collective effect’ is pronounced, can be directly related to the gastropod’s ecology^20,84^: species foraging on solid substrate (e.g. *L. grandis* and *S. zonata*) exhibit central and lateral teeth that can resist relatively high forces due to their capability of relying on one another. Teeth of species foraging on mixed substrate (e.g. *B. grandidieriana*) can resist to less force, but their centrals and laterals are also capable of bending and gaining support. Species feeding on algae from soft substrate (e.g. *C. johnstoni*) possess teeth that could resist to least forces, since they rather failed individually, even though exhibiting very high bending amplitude in the experiment.

Overall, from analysis of morphology^41,42^, breaking stress experiments^20,84^, material property data^19,49,51^, and from consolidated 3D shape and material properties^50^ we were able to identify trophic specialisations to the feeding substrate, but could also propose the terms ‘monofunctional radula’ and ‘multifunctional radula’ for paludomid gastropods^51,84^. A monofunctional radula possesses only teeth that have a high ability to bend and deform and rather collect food particles from the soft substrate. Multifunctional radulae exhibit inner teeth (centrals and laterals) that are stiffer and able to rather loosen food particles from solid or mixed substrate, and outer teeth (marginals), that are flexible and mainly collect the loosened particles.

### Aim of the study

Using our protocol introduced on *S. zonata*^50^, we here apply FEA on 3D modelled radulae, with incorporated material properties, from three additional paludomid species. They forage either on solid (*Lavigeria grandis*), soft (*Cleopatra johnstoni*), or mixed substrate (*Bridouxia grandidieriana*) and possess, as proposed, either a multifunctional (*L. grandis*, *B. grandidieriana*) or a monofunctional radula (*C. johnstoni*). We were able to test our previous hypotheses on functional specialisations of tooth types and trophic specialisations for these species. As the analysed radulae of adult gastropods vary greatly in the size of the whole buccal mass and the individual tooth (e.g., width of the radula ranges from 120 to 350 µm) between species, we here additionally aimed at relating the stress and strain distributions with the tooth sizes by considering the force per volume. For this purpose, we also included results previously obtained on *S. zonata* in the present study. We detected, as expected, that smaller radulae show greater stress and strain in the FEA than larger ones, when each tooth is loaded with the same force. However, the results of the FEA are not fully in congruence with the results from the previous breaking stress experiments^20,84^, indicating that more mechanisms in addition to the size, leading to a resistance to stress and strain, must be present in real radulae.

## Materials and methods

### Specimens, radular size and 3D models

The 3D models, used here for FEA, were already published for visualisation purposes of breaking stress experiments (*Cleopatra johnstoni*, *Bridouxia grandidieriana*, *Lavigeria grandis*) and for conducting FEA (*Spekia zonata*)^50,84^.

**Figure 2:**
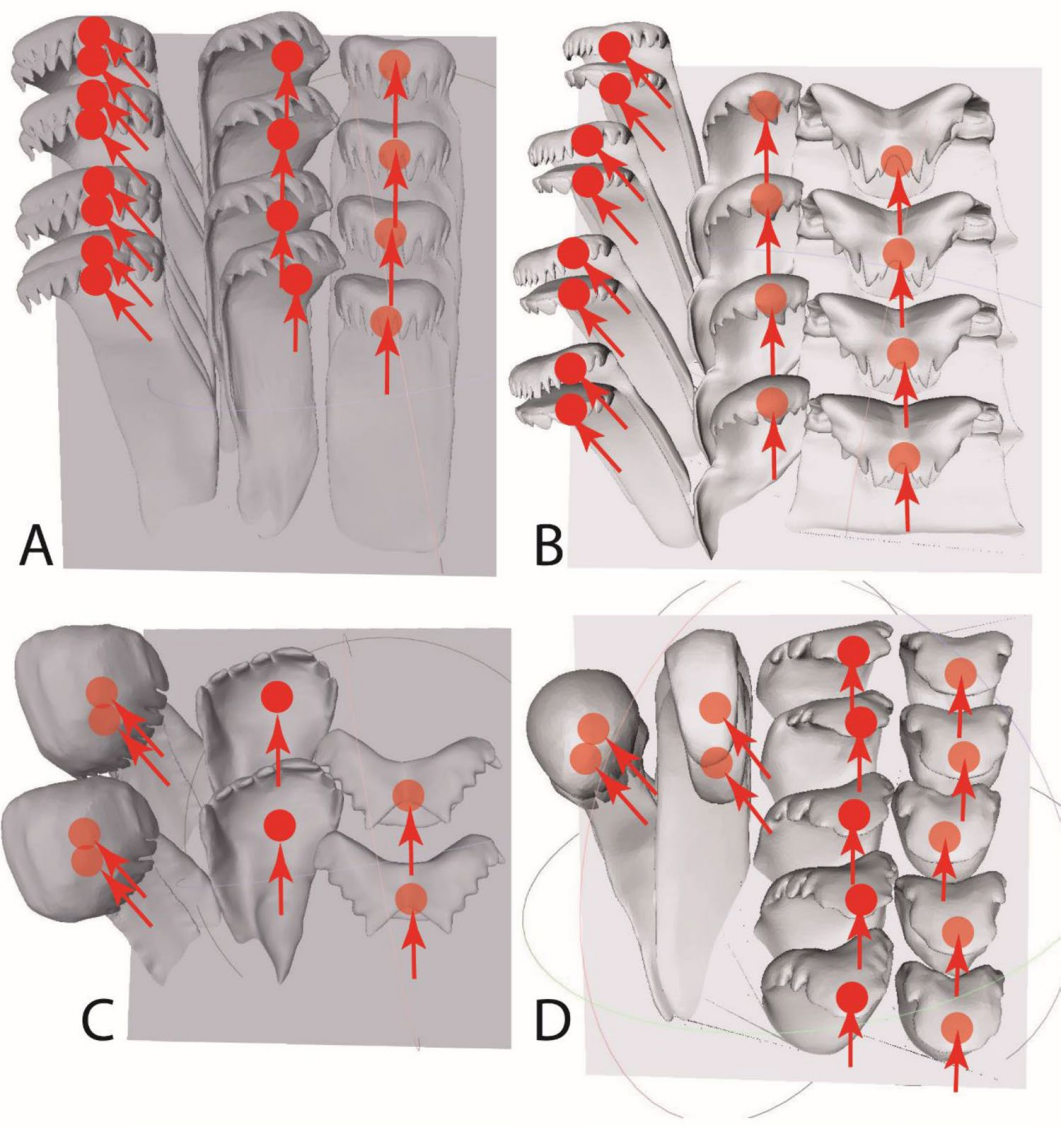
3D models, before creating symmetry, generated in accordance with SEM images from top view (visualized with Meshlab 2016). (**A**) *Cleopatra johnstoni*, (**B**) *Bridouxia grandidieriana*, (**C**) *Spekia zonata*, (**D**) *Lavigeria grandis*. Red dots highlight the areas that were loaded in the FEA, always at the inner area of the cusps. Arrows indicate the direction of force applied (along the anterior–posterior or anterior-medial axis).

**Figure 3:**
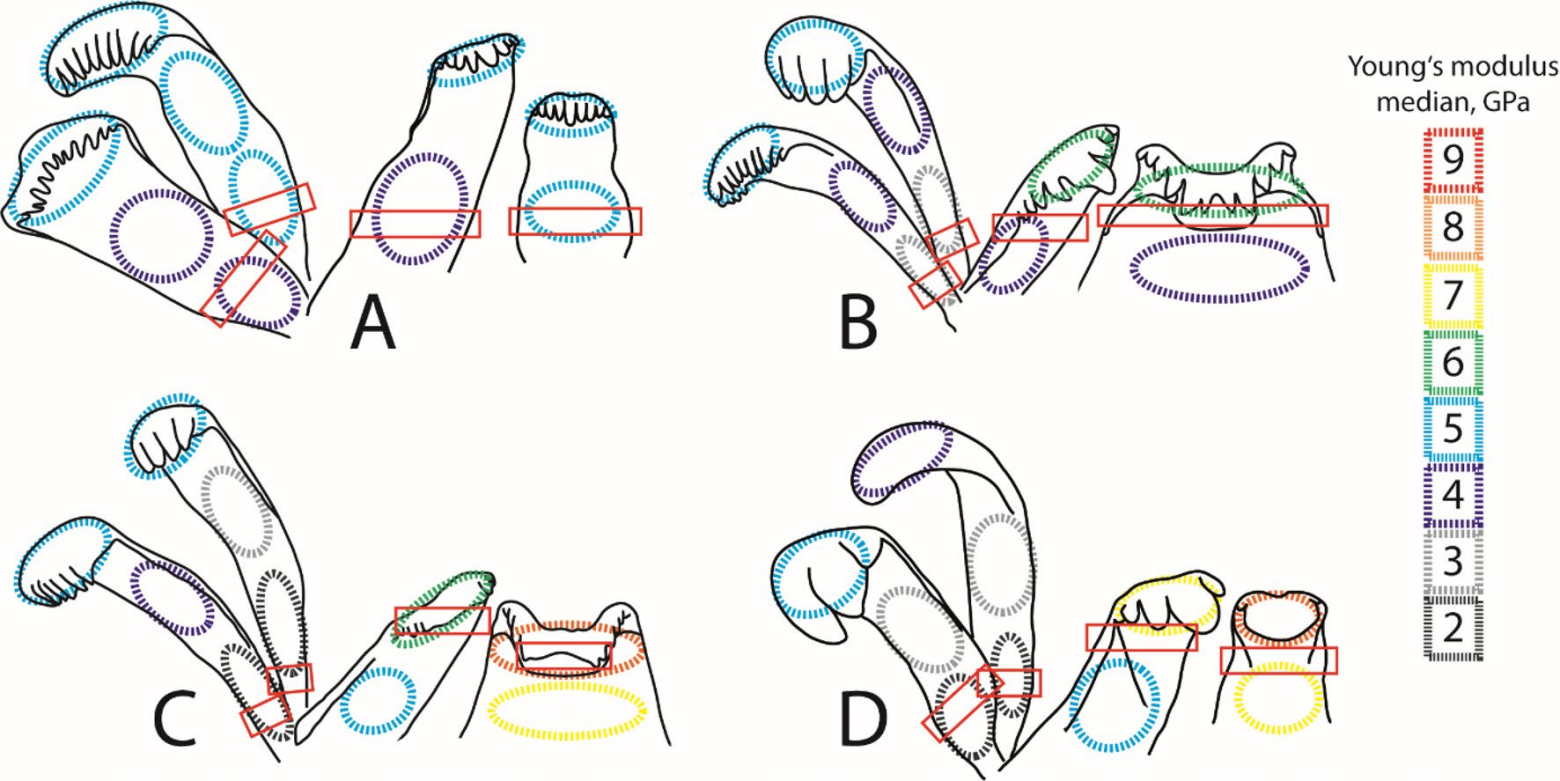
Summary from previous studies on radular teeth failure^20,84^: schematic drawings of radular teeth (adapted from^51^) with median of Young’s modulus, in GPa, plotted on the tooth parts (cusp, stylus, basis) for (**A**) *Cleopatra johnstoni*, (**B**) *Bridouxia grandidieriana*, (**C**) *Spekia zonata*, (**D**) *Lavigeria grandis*. The red boxes highlight the area of failure, if teeth actually broke, during previous breaking stress experiments in wet condition. All marginals broke at their styli close to the basis. In *C. johnstoni* the centrals and laterals also failed at their styli close to the basis. Centrals and laterals in *B. grandidieriana* failed between cusps and styli or teeth bended and failed with the adjacent teeth due to ripping of the membrane. In *S. zonata* and *L. grandis* failure of the centrals and laterals at their denticles or styli was very rarely documented; these teeth rather bended, gained support from adjacent teeth, until all teeth together failed due to rupture of the membrane.

Originally the models (Fig. 2) were created by studying the radulae from adult gastropod specimens (see Fig. 1 for SEM images and Fig. 3 for schematic drawings), that had been either collected by Heinz Büscher in Lake Tanganyika at Zambia, Northern Province, Mibwebwe, in 2019 (*L. grandis* [Smith, 1881]); at Zambia, Northern Providence, Cape Kachese, in 2016 (*B. grandidieriana* [Bourguignat, 1885]); by Frank Riedel at Burundi and by Heinz Büscher at Zambia, Kalambo Falls Lodge, in 2017 (*S. zonata* [Woodward, 1859]) or by Anthony Wilson in Zambia, Lake Mweru, Nchelenge, in 2000 (*C. johnstoni* Smith, 1893). These gastropods were preserved in 70% EtOH and either inventoried at the Zoological Museum Hamburg (ZMH; *B. grandidieriana*: ZMH 119367/999; *L. grandis*: ZMH 154657/999; *S. zonata*: ZMH 150008/999) or the Museum für Naturkunde Berlin (ZMB; *C. johnstoni*: ZMB 220.102; *S. zonata*: ZMB 220.144).

**Figure 4:**
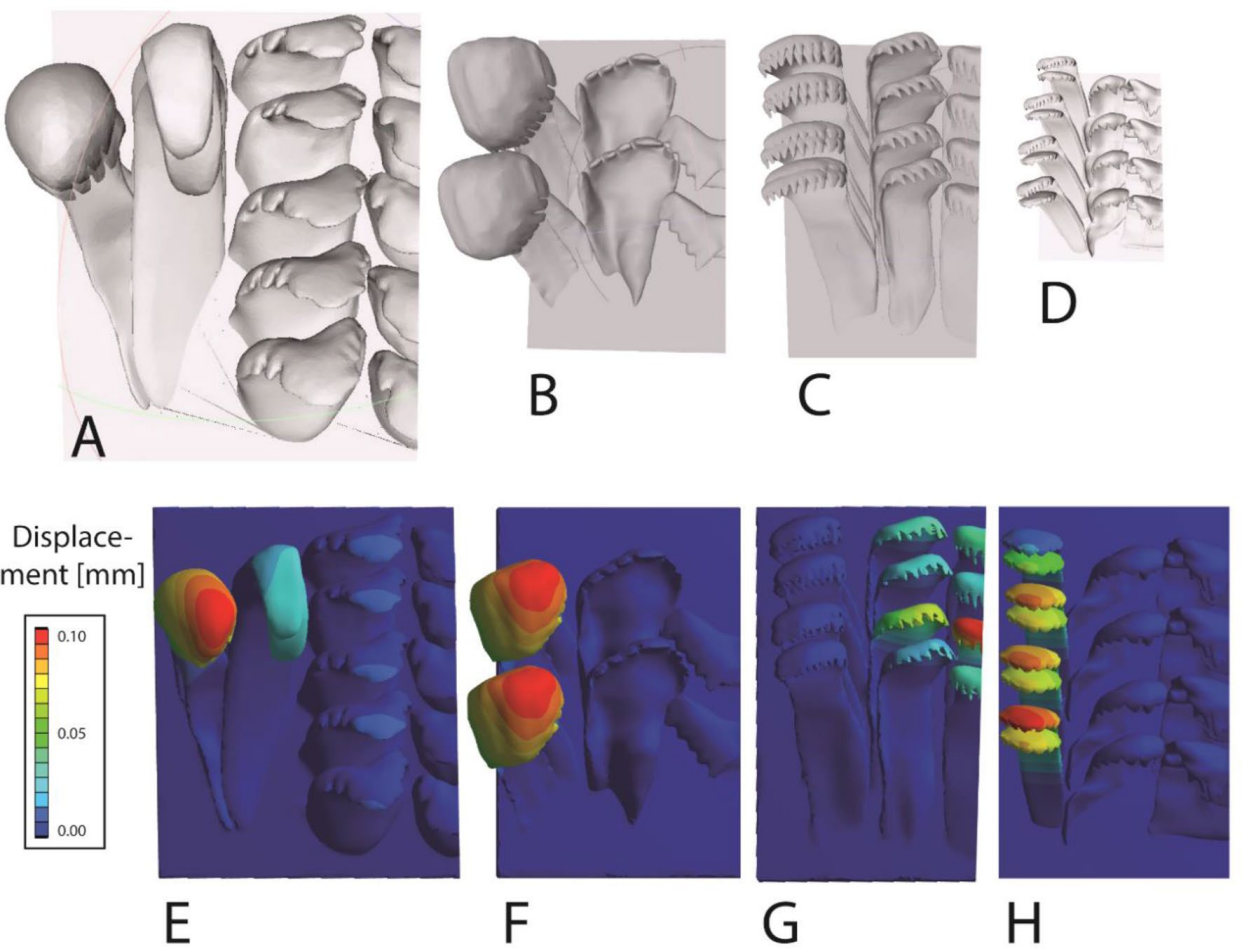
Scaling of the radular teeth as used in the FEA. (**A**, **E**) *Lavigeria grandis*, (**B**, **F**) *Spekia zonata*, (**C**, **G**) *Cleopatra johnstoni*, (**D**, **H**) *Bridouxia grandidieriana*. (**A**–**D**) Models at the same scale. (**E**–**H**) Results from the FEA, displacement after being loaded with the nominal force.

To receive models, two adult specimens per species were dissected, radulae were extracted by tweezers, and cleaned following the protocol of Holznagel^91^. Radulae were first mounted on SEM sample holder, air dried, and documented with the SEM Zeiss LEO 1525 (One Zeiss Drive, Thornwood, NY) (Fig. 1). These images of the two radulae were used for determining the mean width of the radular membrane in the working zone, which was later used for scaling the models (see below). Afterwards, radulae were rewetted with 70% EtOH, loosened from the carbon tape, and manually destroyed by tweezers to receive as many individual teeth as possible. Then, teeth were mounted again on SEM holders in different positions and visualized again by SEM. By this procedure, we received images of individual teeth from different perspectives. With the 3D software Maya 2019 (Autodesk, Inc., San Rafael, USA), one model per tooth type was then formed by hand, always comparing the model with the SEM images to receive ideal 3D teeth. This time-consuming protocol was necessary as we could not receive good models by the µ-CT technique, because tooth structures are too small and of a low contrast. Afterwards, tooth models were first filled, then copied multiple times and some mirrored, to obtain seven teeth per row and overall two tooth rows. Then teeth were assembled and interlocked as observed during breaking stress experiments under load^20,84^. Then, one side of the generated radular model was cut to receive half of a radula. In Maya, all models were sized (Fig. 4) in accordance with the data obtained from SEM images (Fig. 1). Then, the volume of the two half-rows could be read out in Maya. Finally a box, representing the membrane, was modelled for each species and merged with the teeth. Surface irregularities were repaired using Geomagic Wrap 2017 (3D Systems, Inc., Moerfelden-Walldorf, Germany) and models were converted to CAD file format necessary for ANSYS FEA Package, as done previously^50^.

### FEA model

The mechanical properties (Young’s modulus) of the teeth of species studied were identified in previous studies: at the cusps and styli of the lateral and central teeth and on the bases, styli, and cusps of the marginal teeth I and teeth II by nanoindentation^19,49,51^, which was based on the continuous stiffness measurement technique^92^. In the present study, the mean values of the previously measured Young’s moduli (see Table 1) were assigned to the areas of the models, where they were detected (basis, stylus, cusp; see Fig. 3), as it was previously done for *Spekia zonata*^50^. By employing the thermal diffusion method^93^ of the FEA package, values were smoothly diffused through the teeth (see also^94^). Due to the low thickness of the membrane and due to the rapid mechanical changes while drying, we were not able to measure the hardness and elasticity of the membrane by nanoindentation. We therefore assigned the smallest Young’s modulus value, measured for the species’ teeth (following the protocol of^50^), to the membrane of each model (in *Bridouxia grandidieriana*: 3.3 GPa, *Lavigeria grandis*: 2.4 GPa, *Cleopatra johnstoni*: 4.3 GPa, *Spekia zonata*: 2.2 GPa).

**Table 1:** Summary of the preferred feeding substrate with references, the previously assigned feedings substrate categories (mixed, solid, and soft; from^19,51^), and previously determined Young’s moduli (GPa) of the distinct tooth parts. Collection numbers, the quantity of analysed specimens and tooth parts (data from^19,49,51^) are provided for each species studied.

Species	Feeding substrate	Reference for feeding substrate	Feeding substrate category	Summary from previous nanoindentation analysis						Measurements for FEA		
				Collection number	N of analysed specimens	Tooth type	Tooth part	N of analysed teeth	Young’s modulus, GPa, mean	Width of radula, µm mean ± SD	Volume, µm^3^	Nominal force
*Bridouxia grandidieriana* (Bourguignat, 1885)	Sand, plant surface	^68, 95, 96^; personal comment from one collector (Matthias Glaubrecht)	Mixed	ZMH 119367/999	9	Central tooth	Cusp	167	5*.*8	120 ± 7	1,728,000	1.00
							Stylus		4*.*7			
						Lateral tooth	Cusp	176	5*.*6			
							Stylus		4*.*4			
						Marginal tooth I	Cusp	156	4*.*4			
							Stylus		3*.*6			
							Basis		3*.*4			
						Marginal tooth II	Cusp	141	4*.*5			
							Stylus		3*.*4			
							Basis		3*.*3			
*Lavigeria grandis* (Smith, 1881)	Rock	^68, 69, 97, 98^	Solid	ZMB 220.018, ZMH 150020/999	6	Central tooth	Cusp	84	8*.*1	350 ± 11	42,882,000	8.51
							Stylus		6*.*8			
						Lateral tooth	Cusp	111	6*.*5			
							Stylus		5*.*1			
						Marginal tooth I	Cusp	106	4*.*6			
							Stylus		3*.*3			
							Basis		2*.*4			
						Marginal tooth II	Cusp	102	4*.*4			
							Stylus		3*.*4			
							Basis		2*.*5			
*Cleopatra johnstoni* Smith, 1893	Sand, Mud	Unpublished work, personal comment from one collector (Matthias Glaubrecht)	Soft	ZMB 220.102b	8	Central tooth	Cusp	151	4*.*7	220 ± 9	10,646,000	3.34
							Stylus		4*.*6			
						Lateral tooth	Cusp	123	4*.*6			
							Stylus		4*.*3			
						Marginal tooth I	Cusp	101	4*.*6			
							Stylus		4*.*6			
							Basis		4*.*5			
						Marginal tooth II	Cusp	143	4*.*8			
							Stylus		5*.*1			
							Basis		4*.*6			
*Spekia zonata* (Woodward, 1859)	Rock	^61, 68, 69, 95–101^; personal comment from collectors (Heinz Büscher and Matthias Glaubrecht)	Solid	ZMB 220.077, ZMB 220.143, ZMH 150008/999	7	Central tooth	Cusp	110	8*.*1	250 ± 10	15,725,000	4.36
							Stylus		6*.*7			
						Lateral tooth	Cusp	112	5*.*8			
							Stylus		5*.*0			
						Marginal tooth I	Cusp	57	4*.*9			
							Stylus		4*.*1			
							Basis		2*.*2			
						Marginal tooth II	Cusp	60	4*.*6			
							Stylus		3*.*3			
							Basis		2.4			

Geometrical variables of radulae (width of radulae and volume of the model) necessary to determine the nominal force for each species.

*N* quantity, *SD* standard deviation.

**Figure 5:**
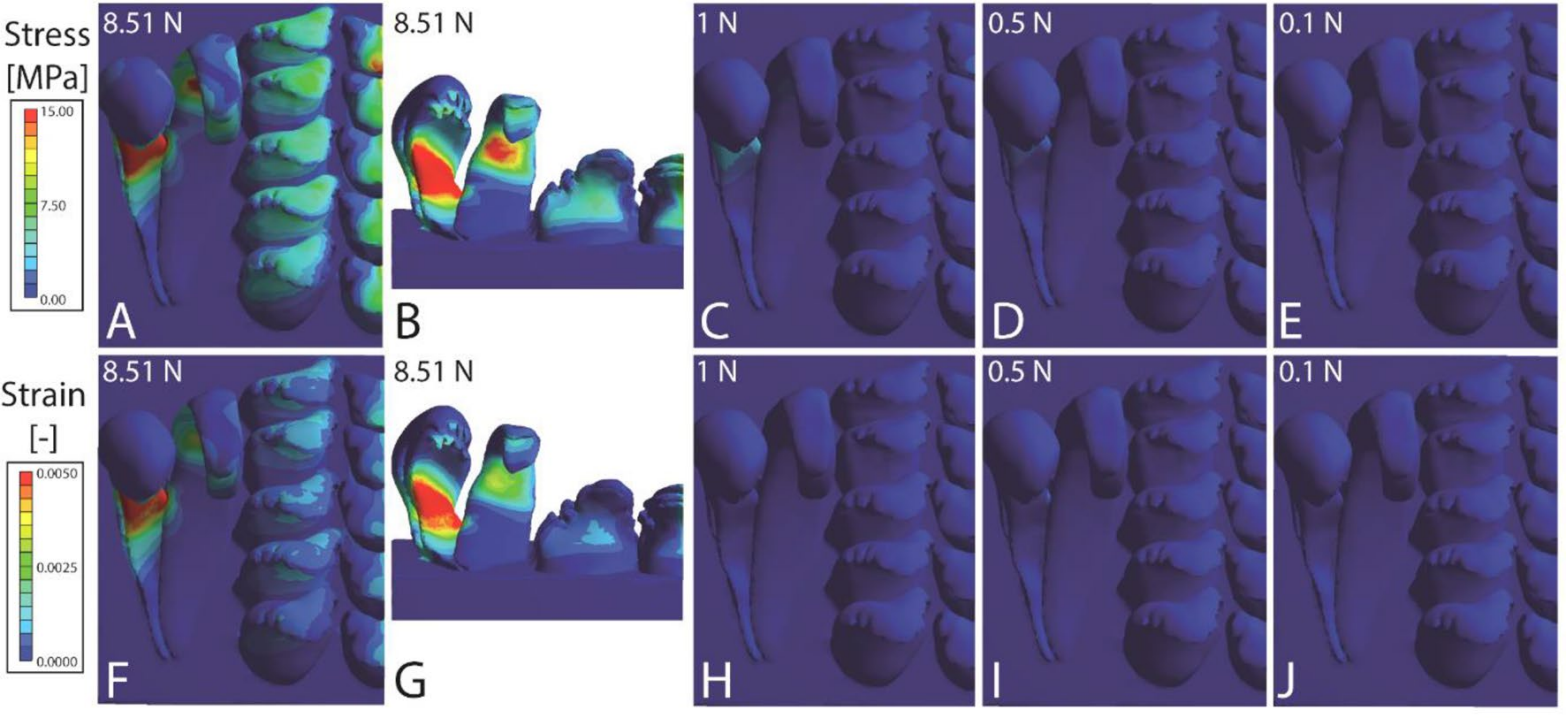
Results from the FEA for *Lavigeria grandis*. (**A**–**E**) Stress and (**F**–**J**) strain. (**A**, **B**, **F**, **G**) Loaded with nominal force (8.51 N); (**C**, **H**) with 1 N; (**D**, **I**), with 0.5 N; (**E**, **J**) with 0.1 N. The scaling of stress and strain is identical in Figs. 5, 6, 7 and 8.

**Figure 6:**
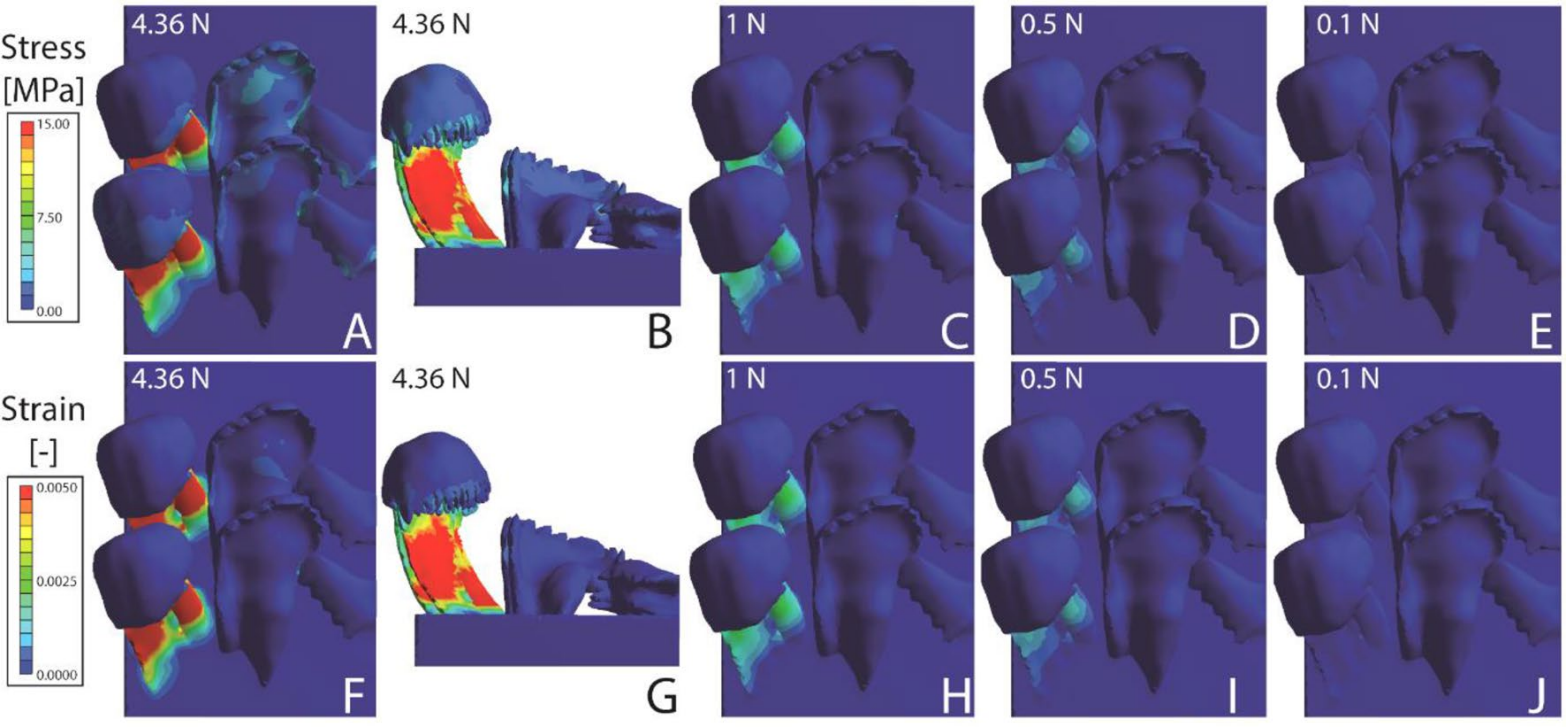
Results from the FEA for *Spekia zonata*. (**A**–**E**) Stress and (**F**–**J**) strain. (**A**, **B**, **F**, **G**) Loaded with nominal force (4.36 N); (**C**, **H**) with 1 N; (**D**, **I**), with 0.5 N; (**E**, **J**) with 0.1 N. The scaling of stress and strain is identical in Figs. 5, 6, 7 and 8.

**Figure 7:**
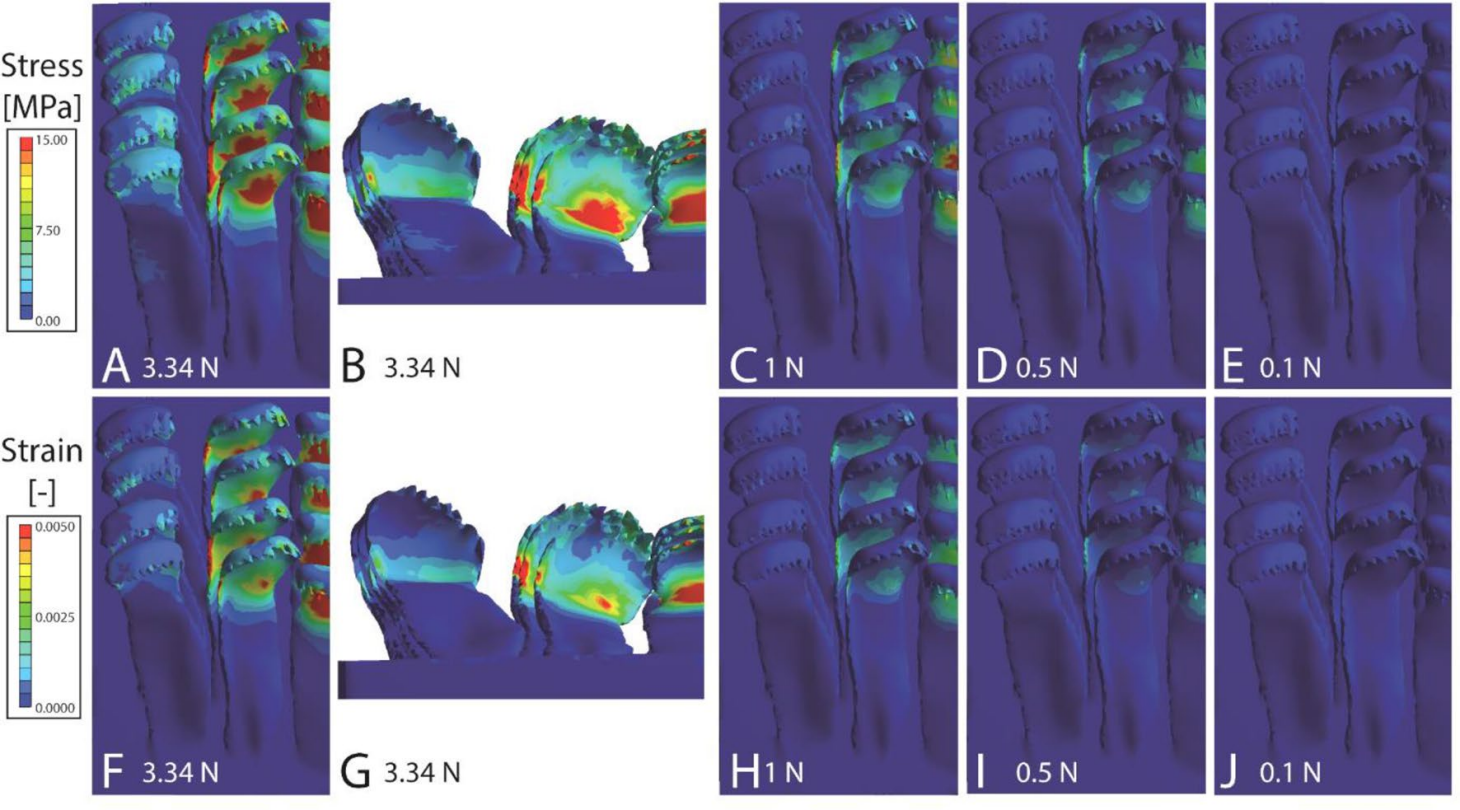
Results from the FEA for *Cleopatra johnstoni*. (**A**–**E**) Stress and (**F**–**J**) strain. (**A**, **B**, **F**, **G**) Loaded with nominal force (3.34 N); (**C**, **H**) with 1 N; (**D**, **I**) with 0.5 N; (**E**, **J**) with 0.1 N. The scaling of stress and strain is identical to those in Figs. 5, 6, 7 and 8.

**Figure 8:**
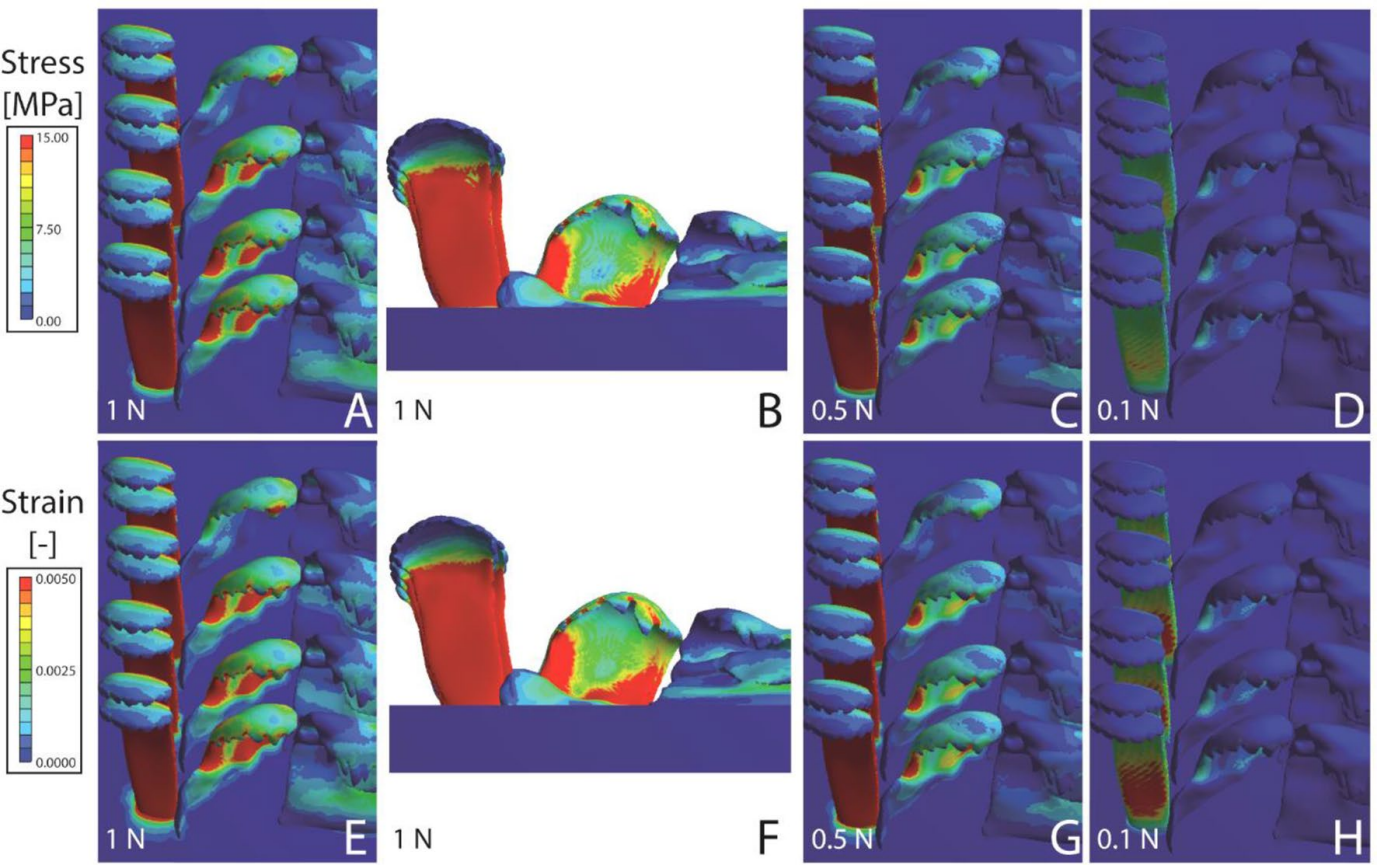
Results from the FEA for *Bridouxia grandidieriana*. (**A**–**E**) Stress and (**F**–**J**) strain. (**A**, **B**, **E**, **F**) Loaded with nominal force (1 N); (**C**, **G**) with 0.5 N; (**D**, **H**) with 0.1 N. The scaling of stress and strain is identical in Figs. 5, 6, 7 and 8.

A structural static analysis was performed employing the FEA package ANSYS 17.1 (Ansys, Canonsburg, USA) in a Dell Precision Workstation T7820 with 64 GB RAM. The radulae were meshed using the ANSYS mesh module with an adaptive mesh of hexahedral elements^102^ resulting in about 100,000 elements per model. As result we received qualitative stress and strain distribution plots. Areas of high local stress or strain are coloured in red, of no stress or strain in blue, green and yellow are intermediate (please see scales in the Figs. 5, 6, 7, 8). We tested different biomechanical scenarios in function of the value and the direction of the force.

#### Direction of force

We applied force always to the cusps of each central and lateral tooth along the anterior-posterior axis of the radula (see Fig. 2). For the marginal teeth, we tested two directions of forces: along the radular anterior-posterior and anterior-medial axes. We detected, as in the previous study on *Spekia zonata*^50^ that stress and strain values are smaller, when loaded along the anterior-medial axis. This, together with recent findings obtained from building and moving a physical radular model for *S. zonata*^103^, suggests that the marginal teeth rather perform an ‘inward raking’ and are loaded along the radular anterior-medial axis. Therefore, we have chosen this direction of force for both marginal teeth.

#### Nominal force

A load of 1 N per tooth was set for the smallest 3D model of *Bridouxia grandidieriana*. To compare the different models, the nominal forces for the other species (e.g. *Cleopatra* *johnstoni*) were determined by calculating nominal_force^*C. johnstoni*^ = (volume^*C. johnstoni*^/volume^*B. grandidieriana*^)^2/3^ (see Table 1) using *B. grandidieriana* as a reference. This is the usual procedure to scaling the models to comparable size: keep the differences in the FEA model and apply an appropriate force that generates the effect of removing the influence of the size^104^.

#### Other loading conditions

Additionally we loaded each tooth with 1 N, 0.5 N, and 0.1 N. These force values were chosen since in the previous breaking stress experiments tooth failure was observed between 1 and 0.5 N, depending on the species^20,84^.

Finally, all the models were fixed in the posterior part of the membrane and we included as a fixed displacement the contact areas on the superior marginal, central and lateral and tooth with a hypothetic new row of teeth above.

### Ecology

The information on the preferred feeding substrate was summarized from the literature or from personal comments of collectors in the field (see Table 1). *Lavigeria grandis* and *Spekia zonata* forage on algae attached to rocks (solid substrate), *Cleopatra johnstoni* forages on algae that cover sand or mud (soft substrate), and *Bridouxia grandidieriana* on algae from various surfaces: sand, mud, plant surface (mixed substrate).

## Results

### Morphology of teeth and radular size

The detailed morphology of teeth was previously described in^19^. All marginal teeth (see Figs. 1, 2, 3 and 4 for tooth images, schematic drawings, and 3D models) are of rather slender shape with elongated styli and possessing a cusp with multiple and rather fine denticles (except for the inner marginal tooth of *Lavigeria grandis*, possessing no denticles, see Fig. 1J). Centrals and laterals of *Cleopatra johnstoni* are of similar shape to the marginal teeth (Fig. 1E, F). Central and lateral teeth of *L. grandis* and lateral teeth of *Spekia zonata* possess one broad and prominent central denticle on the cusp, situated on elongated styli (Fig. 1I, J). The central tooth of *S. zonata* is rather broad with only few small denticles (Fig. 1I). *Bridouxia grandidieriana* possess a broad central tooth with fine denticles and a rather slender lateral tooth with elongated stylus and cusp exhibiting one prominent denticle and multiple finer ones (Fig. 1G, H). The radulae from adult *B. grandidieriana* specimens are the smallest ones (mean width ± standard deviation; 120 ± 7 µm), followed by *C. johnstoni* (220 ± 9 µm), *S. zonata* (250 ± 10 µm), and finally *L. grandis* with the largest radulae (350 ± 11 µm).

### Results of the FEA

#### Displacement

In *Lavigeria grandis*, *Spekia zonata*, and *Bridouxia grandidieriana* only the marginals, in *Cleopatra johnstoni* laterals and centrals are displaced during load, whereas all other teeth do not show significant values of displacement (Fig. 4).

#### Stress and strain in each species and tooth type

The results of the FEA are sorted to species in Figs. 5, 6, 7 and 8. In *Lavigeria grandis* (Fig. 5), marginal tooth II experiences the highest stress and strain, followed by marginal tooth I, lateral tooth, and finally central tooth. Here the marginal teeth show the highest values of both parameters on their styli, the lateral teeth on their cusps and styli, and the central teeth on their central denticle of the cusp. In *Spekia zonata,* marginals show very high stress/strain concentrations on the styli of the marginal teeth, and only little stress/strain on the lateral tooth cusps and the anterior margin of the central tooth cusps (Fig. 6). In *Cleopatra johnstoni*, we observed a completely different pattern, here the highest stress and strain were obtained for central teeth, followed by the lateral teeth, and finally both marginal teeth (Fig. 7). Centrals and laterals show high concentrations of stress and strain on the posterior part of the cusp; the marginals experience stress and strain at the medial and anterior area of the cusps. In *Bridouxia grandidieriana*, the marginal teeth show the highest concentration of stress and strain at the whole length of their styli, both parameters decrease dramatically from the stylus to cusp and basis across a very small area (Fig. 8). The lateral teeth experience highest stress and strain at their inner and outer edges of the styli and on their central denticle of the cusp. The central teeth show stress and strain concentrations on their basis and the medial part of the cusp.

#### Stress and strain for each loading condition

When all models are loaded with the nominal force (forces are corrected for the radular size) the radula of *Bridouxia grandidieriana* possess the largest area with high concentrations of stress and strain, followed by *Cleopatra johnstoni*, *Spekia zonata*, and finally *Lavigeria grandis* (Figs. 5, 6, 7, 8). With decreasing load in each species, stress and strain concentrations are reduced. *L. grandis* shows very little stress and strain when loaded with 1 N, *S. zonata* and *C. johnstoni* little stress and strain when loaded with 0.5 N, whereas *B. grandidieriana* still experiences higher stress and strain even when loaded with 0.1 N.

## Discussion

### Distribution of stress and strain

When models are loaded with the nominal force (force corrected for radular size) in the FEA, we can directly compare effects on stress and strain distribution between species and between tooth types (Figs. 5, 6, 7, 8). When models were loaded with 1 N, 0.5 N, and 0.1 N (forces are not corrected for radular size), similar to the situation in the breakings stress experiments, we can, in contrast, directly see the effect of the radular size on the stress/strain distribution.

Overall, as determined in our previous FEA on the teeth of *Spekia zonata*^50^, the simulated stress and strain under nominal force can be explained by the shape of the teeth and the local mechanical properties that were analysed in previous studies^49,51^. The simulations of the mechanical behaviour are, in most cases, similar to the observed ones, determined by previous breaking stress experiments under wet condition (^20,84^; see Fig. 3 for the areas of failure in the experiment):

Thick, short, and broader teeth, as the centrals and laterals of *S. zonata* and *Lavigeria grandis*, are not as prone to deformation and do not show areas of high local stress, which can enable force transmission to the ingesta (see also^26,89^). These teeth are additionally stiffer, leading to a high ability of the material to transmit forces (for the relationship between Young’s modulus and force transmission see e.g.^105–108^), supporting puncture mechanics and the resistance to failure (see e.g.^109^; review on puncture mechanics see^110^). In our breaking stress experiments these teeth could resist higher forces (mean ± standard deviation; *S. zonata*, laterals: 799.83 ± 313.47 mN, centrals: 979.50 ± 381.14 mN; *L. grandis*, laterals: 270.95 ± 87.49 mN, centrals: 700.90 ± 255.52 mN) and were less prone to failure.

The softer, longer, thinner, and slender teeth, in contrast, as the marginals of *S. zonata* and *L. grandis*, the laterals and marginals of *Bridouxia grandidieriana*, and the centrals and laterals of *Cleopatra johnstoni*, experience higher strain and stress in the FEA. In our previous breaking stress experiments, these teeth could resist less force, showed a high ability of bending, and usually failed (between 83 and 272 mN, depending on the tooth type and species). Overall, models as well as real teeth (a) can deform more easily during interaction with the ingesta, (b) show areas of high stress, and (c) are more prone to failure.

However, even though central teeth of *B. grandidieriana* are relatively thin, they show lesser concentrations of stress and strain in the simulations and additionally could resist higher forces (329.11 ± 128.06 mN) than marginals (96.08 ± 13.33 mN) in previous breaking stress experiments. This could be explained by their mechanical properties, as they have a higher Young’s modulus than the marginals (Fig. 3), and by the morphology of their basis. They are broader thus and possess a large attachment area with the membrane, enabling better stress redistribution (see also^42^). The breaking stress experiments also revealed that the centrals can resist to similar force than the laterals (315.31 ± 104.99 mN), even though the latter are narrower and have similar mechanical properties. In our FEA however the laterals are more affected from stress and strain than the central teeth (Fig. 8). This inconsistency between the FEA and experiments could be explained by the ability of the wet lateral teeth to bend and rely on the lateral teeth from the adjacent rows, gaining support (for the importance of tooth-tooth interaction see also^86–90^). This ‘collective effect’, involving dynamic radular models, is however difficult to simulate in the FEA.

Within each heterogeneous tooth (with gradients in Young’s modulus), the areas of high local stress and strain also correspond to the values of the Young’s modulus. Additionally, the simulated mechanical behaviours of the tooth areas usually reflect real mechanical behaviour observed in breaking stress experiments. Areas that are rather soft, as the styli and bases of the marginals in *B. grandidieriana*, *S. zonata*, and *L. grandis* and of the laterals in *B. grandidieriana*, exhibit a high ability to deform both in simulations and experiments. In docoglossan teeth, this bending behaviour of the stylus has also been previously simulated by the FEA^58^. In our simulations, these areas additionally show high local stress, which corresponds to the areas of failure in the experiment (Fig. 3). In contrast, the stiffer cusps did not deform as much as in the experiment and did not show high local stresses in our FEA. For radular teeth, the importance of the heterogeneous distribution of material properties was previously also determined in docoglossan teeth of *Patella* and Polyplacophora; here^7^ detected that the tooth’s part, interacting in the ingesta, is harder and stiffer, whereas the underlain parts are softer and more flexible (see also^58^ for the flexibility of the stylus). The co-appearance of harder and softer layers probably leads to a reduction of abrasion in the radular cusps^7,46^ as observed in other structures as well (e.g.^54,111^). The flexibility of both the stylus and basis probably serves as a shock absorber, when interacting with obstacles (see also^58,90^), a mechanism also previously reported from other biological structures (e.g.^112–114^).

For the homogeneous teeth (with similar Young’s moduli) of *C. johnstoni* we previously detected, that their force-resistance, determined by breaking stress experiments, is the highest in centrals (350.89 ± 49.44 mN), followed by laterals (170.46 ± 32.30 mN), and finally marginals (136.75 ± 16.50 mN). This is contrary to the distributions of stress and strain, obtained from the FEA (here stress and strain are low in the marginals and high in the centrals and laterals). This can be explained by the specific arrangement of teeth: in our models the outer marginal tooth embraces the inner, smaller one, leading to a reduction of high local stress and to a deceased ability of both teeth to deform together. This is the configuration that can be often observed in the SEM (Fig. 1E). In our previous breaking stress experiments, radulae were extracted from the specimens, taped onto glass objects slides, and teeth were stroked into the proposed feeding position. During this latest step, marginal teeth were unfortunately separated in many cases, thus breaking stress experiments were performed with individual and not with naturally interlocking teeth. If and to which extend embracing teeth can resist to higher force should be studied in further experiments. Within *C. johnstoni*’s teeth, we detected high stress and strain at the area between each cusp and stylus, which is in contrast to the experiments, where failure occurred between the stylus and basis (Fig. 3). This can be also explained by the position of teeth in the simulations, as each stylus has a relatively large area of contact with the adjacent stylus from the same tooth type, leading to a distribution of stress across the styli to the underlain radular membrane, but also reducing the stylus’ ability to deform.

When stresses are not corrected for size, we detect, as expected, that smaller radulae show more stress and strain than larger ones, with *B. grandidieriana* showing the highest local concentrations of stress/strain, followed by *C. johnstoni*, *S. zonata*, and finally *L. grandis* with the lowest ones. Even though the FEA does not allow direct conclusions about failure behaviour or force-resistance of structures, we would suggest the following relationship between the breaking forces, determined in the experiments, with the stress simulations under the defined loads (1 N, 0.5 N, 0.1 N): in the experiment, *C. johnstoni*’s teeth were able to resist to maximal 400 mN (comparable to FEA, loaded with 0.5 N), *B. grandidieriana*’s to 460 mN (comparable to FEA, loaded with 0.5 N), *L. grandis*’s to 956 mN (comparable to FEA, loaded with 1 N), and *S. zonata*’s to 1360 mN (comparable to FEA, loaded with 1 N). When the stress scales of the FEA models are considered, structural failure should actually occur, when areas of the modelled teeth experience between 1 to 6 MPa. However, as mentioned above, *S. zonata* is able to resist to the highest forces in real experiments, followed by *L. grandis*, *B. grandidieriana*, and finally *C. johnstoni*. This already indicates that besides the parameter size, other factors seem to be important to reduce stress and strain in living radula. Our here presented models have distinct local Young’s moduli, but are modelled as filled solid bulk materials. Real teeth are, however, composed of fibres and their arrangement, size, and density seem to contribute to the reinforcement of the tooth itself, as it was detected for limpet and chiton teeth^45–47,115–120^. These parameters, however, await further investigations in the paludomid teeth.

### Functional and trophic specialisations of tooth types

Padilla^89^ summarized previous approaches on radular function and proposed new avenues to gain deeper insight into its functionality and in general to molluscan ecology. She highlighted the importance of the 3D shape, material properties, and interaction of teeth.

By the here presented results of the FEA, that include these parameters, we were able to verify previous hypotheses about tooth functionalities in paludomid gastropods. In soft substrate feeders, all teeth are rather used for collecting particles (‘monofunctional radula’ ^51,84^), whereas in mixed and solid substrate feeders, the centrals and laterals rather loosen food from the substrate, and the marginals collect the particles afterwards (‘multifunctional radula’ ^49,51,84^).

This is supported, as mentioned above, by the simulated mechanical behaviour of teeth under nominal force (see also^50^ for *S. zonata*): the centrals and laterals of *L. grandis* and *S. zonata* are rather capable of transferring forces without deformation. The marginals show, in contrast, a high capability of bending at the basis and the stylus, which results in the reduction of failure, but also does not facilitate a direct transfer of forces from the radula to the food. They probably gather the loosened particles in form of an ‘inward raking’ during retraction of the radula from the ingesta (see^28^). This hypothesis is also supported by another previous approach, involving a physical radular model of *S. zonata*^103^. In that study, we performed dissections of adult specimens, extracted the whole buccal mass with the musculature and radula, documented the anatomy, and mimicked the structures by 3D printing and assembly of fabrics. With this approach, we were able to build the first, relatively simple, but movable radular model. By the manipulation of the radular supporting structures of the model the interaction and ranges of motion of the radular structures could be documented. Hereby we found that the marginal teeth are flexed as consequence from a rotation of the underlain buccal mass musculature and perform a raking motion from the middle of the radula to the outer edges, an ‘inward raking’.

For the mixed substrate feeder *B. grandidieriana* we detect that the central teeth are rather capable of transferring forces, similar to the solid substrate feeders. However, the laterals are more affected from stress and strain, which indicates that they are probably not capable of transferring forces as efficient as the central teeth. So, potentially the lateral teeth are rather involved in gathering particles, similar to the marginal teeth.

A high ability of bending and the presence of areas of high local stress, leading to a higher risk of breaking, was also simulated by the FEA on the centrals and laterals of *C. johnstoni.* For taenioglossan radulae, it was previously hypothesized that the central teeth are rather used for gathering food^28,121,122^, which seems to be the case for *C. johnstoni,* but, as mentioned above, not for *L. grandis* and *S. zonata*. Thus, the functionality of certain tooth types varies greatly between species. We however, also detected by the FEA that the marginals of *C. johnstoni*, when arranged in an embracing position, show less stress and strain. This indicates that in this species marginals are potentially more effective in forcefully loosening algae, which was previously also proposed for the marginal teeth of *Littorina*^123^.

As mentioned above, we determined by the FEA that with increasing radular size, stress and strain decreases. However, in previous breaking stress experiments, teeth of *S. zonata* could resist higher forces than those of *L. grandis,* and *B. grandidieriana* higher forces than *C. johnstoni*^84^. We therefore propose that the observed size differences between the species studied are the result of adaptations to distinct algae cover types rather than an assistance in foraging on the same algae cover, but with a greater food loosening ability due to larger contact areas between tooth and ingesta. E.g. *S. zonata* could potentially forage on (a) tougher or harder biofilms or (b) thinner biofilms resulting in a more frequent interaction with the rock underneath the biofilm than *L. grandis*. To test this hypothesis, however, the identification, sampling, and finally mechanical testing of the biofilm and algal coverage in Lake Tanganyika is necessary since data on this is lacking.
